# Beyond monospecificity in systemic lupus erythematosus: Emerging roles of bispecific and trispecific antibodies

**DOI:** 10.1177/09612033261493822

**Published:** 2026-10-07

**Authors:** Simone Negrini, Stefania Nicola, Luisa Brussino

**Affiliations:** 1 Department of Medical Sciences, 9314 University of Turin, Turin, Italy; 2 Immunology and Allergy Unit, AO Ordine Mauriziano di Torino, Turin, Italy

**Keywords:** systemic lupus erythematosus, lupus nephritis, bispecific antibodies, trispecific antibodies, T-cell engagers, B-cell therapy

## Abstract

**Purpose:**

Targeted biologic therapy has changed the management of systemic lupus erythematosus (SLE), including lupus nephritis (LN), but incomplete response, relapse, glucocorticoid dependence, and refractory organ disease remain common. This article discusses whether bispecific and trispecific antibodies may help move lupus therapy beyond conventional monospecific blockade.

**Key Messages:**

Multispecific antibodies are not a single therapeutic class. Their clinical relevance depends on mechanism: dual-pathway blockade, interruption of B-T-cell collaboration, inhibitory non-depleting B-cell silencing, T-cell engager-mediated depletion of B-cell or plasma-cell compartments, and future trispecific immune-reprogramming concepts. Direct lupus evidence remains limited. Obexelimab, which co-engages CD19 and the inhibitory receptor FcγRIIb, provides the most informative randomised SLE dataset, but its phase 2 trial did not meet the primary endpoint and biomarker-defined signals remain hypothesis-generating. Teclistamab, a BCMA × CD3 T-cell engager, offers a provocative single-case proof of concept in refractory SLE with active LN, but also illustrates risks of cytokine release syndrome, infections, and hypogammaglobulinaemia. Rozibafusp alfa, which targets ICOSL and BAFF, illustrates the biological appeal, and clinical difficulty, of rational dual-pathway blockade in SLE.

**Conclusions:**

Multispecific antibodies may become useful in lupus if their depth of immune intervention can be matched to disease phenotype, safety risk, and trial design. Their future role will depend on lupus-specific evidence, biomarker-guided selection, and careful positioning between conventional biologics and cellular immune-reprogramming strategies.

## Why move beyond monospecificity in SLE?

Systemic lupus erythematosus (SLE) has entered an era in which targeted therapy is no longer a theoretical aspiration, because BAFF inhibition, type I interferon blockade, and B-cell-directed approaches have shown that clinically meaningful disease modification is possible beyond conventional immunosuppression.^1–6^ Yet many patients continue to experience incomplete response, relapse, persistent serological activity, glucocorticoid exposure, or progressive organ damage, particularly in lupus nephritis (LN).^1–4^ The gap between measurable improvement and durable immune control therefore remains substantial.^1–4^

This gap reflects disease biology rather than therapeutic failure alone.^4^ SLE is sustained by interacting circuits involving autoreactive B cells, T-cell help, plasmablast and plasma-cell differentiation, type I interferon amplification, cytokine networks, tissue-specific injury pathways, and genetic and environmental heterogeneity.^4^ Monospecific biologics have validated individual nodes within this network, but they have also exposed the limits of one-target intervention.^1–4^ Belimumab confirmed BAFF as a clinically relevant pathway in SLE, including LN, but BAFF blockade alone does not fully extinguish autoreactive humoral immunity.^1,2^ Anifrolumab established type I interferon signalling as a therapeutic target in active SLE, but interferon blockade does not directly remove the B-cell and plasma-cell compartments that maintain autoantibody production.^3,4^ Anti-CD20 strategies have strengthened the rationale for B-cell targeting, including in LN, but conventional depletion does not consistently translate into durable treatment-free disease control.^4–6^

The question is therefore not whether monoclonal antibodies work in lupus, but whether the next therapeutic step should remain monospecific.^1–4,7–9^ Bispecific and trispecific antibodies challenge the assumption that one molecule must equal one target and one mechanism.^7–9^ Depending on their architecture, they can block two pathogenic pathways, interfere with B-T-cell crosstalk, deliver inhibitory signals to B cells, redirect endogenous T cells against pathogenic immune compartments, or combine broader targeting and effector engagement in a single construct.^7–20^ Their appeal in SLE is conceptual as much as technological: they may allow therapeutic design to better match disease architecture.^4,7–9^

This article focuses on the therapeutic logics most relevant to SLE, including LN: dual-pathway blockade, disruption of B-T-cell collaboration, inhibitory non-depleting B-cell strategies, T-cell engager-mediated depletion, and future trispecific immune-reprogramming concepts.^10–14,16–20^ The evidence remains early and uneven.^12–14,21^ Obexelimab, which co-engages CD19 and FcγRIIb, provides the most informative randomised lupus dataset, but did not meet its primary endpoint.^12^ Teclistamab, a BCMA × CD3 T-cell engager, offers a provocative single-case proof of concept in refractory SLE with active nephritis, but also illustrates the toxicity of deeper plasma-cell-directed intervention.^13^ Rozibafusp alfa, which targets ICOSL and BAFF, and other dual-pathway approaches show biological plausibility, but clinical superiority in SLE remains unproven.^10,16,21^ Multispecific antibodies are therefore not simply “next-generation monoclonals”; they represent an attempt to solve a problem that monoclonals helped define: targeted therapy works in lupus, but single-target intervention often does not match the layered structure of the disease.^1–4,7–9^ The central issue is which multispecific strategy fits which lupus phenotype, at what disease stage, and with what acceptable immune cost.^4,12–14,21^

## Why format matters

The value of multispecific antibodies in SLE does not lie only in the number of targets they bind, but in how molecular format changes therapeutic intent.^7–9^ A bispecific antibody can function as a dual blocker of soluble mediators, a modulator of immune-cell crosstalk, an inhibitory signal-delivering molecule, or an off-the-shelf cell-depleting platform.^7–19^ These are fundamentally different therapeutic propositions despite sharing the label of bispecificity.^7–9^

This distinction matters in lupus because the margin between efficacy and excess immune suppression is narrow.^4,12–14^ In oncology, aggressive immune engagement may be acceptable if it produces major antitumor activity.^7,8^ In SLE, including LN, the target is a dysregulated immune system that must remain competent enough to protect against infection.^4,12–14^ The central design question is therefore how to calibrate depth, reversibility, and selectivity, not simply how to increase potency.^4,7–9^

IgG-like and Fc-free formats illustrate the point.^7–9^ IgG-like multispecifics retain an Fc-containing antibody architecture, which usually provides longer half-life, familiar pharmacokinetics, and compatibility with intermittent dosing.^7–9^ These features are attractive in chronic autoimmune disease, where repeated outpatient administration and predictable exposure matter.^7–9^ Smaller Fc-free constructs may improve immune-cell proximity or tissue penetration, but often have shorter half-lives and may require more intensive dosing strategies.^7–9,19^ Blinatumomab, the canonical CD19×CD3 BiTE, illustrates this trade-off because it can redirect T cells against B cells, but its short half-life and infusion requirements are not naturally suited to routine chronic autoimmune care.^7–9,19^

Format also determines mechanism.^7–9^ Dual-antagonist constructs such as BAFF × IL-17 or ICOSL × BAFF are designed primarily to suppress parallel pathogenic pathways.^10,11,15,16^ In this setting, valency and avidity matter because the molecule must neutralise soluble mediators or receptor-ligand interactions efficiently.^7,8,9,15,16^ T-cell engagers require a different design logic because activity depends on bringing CD3-expressing T cells into contact with B-cell or plasma-cell-lineage targets and forming an artificial cytotoxic synapse.^7–9,13,14,19^ In these constructs, CD3 affinity, geometry, and target-antigen density may influence both efficacy and cytokine release syndrome.^7–9,13,14^

Inhibitory non-depleting B-cell strategies introduce a third logic.^12,17^ Obexelimab co-engages CD19 and FcγRIIb to suppress B-cell activation without inducing broad cytotoxic depletion.^12,17^ Trispecific antibodies extend the framework further, but in lupus they remain a future-facing concept rather than a clinically established strategy.^7–9,20^ Their potential value is the ability to combine targeting, effector engagement, and modulatory control within one molecule, whereas their risk is additional complexity in manufacturability, pharmacokinetics, immunogenicity, and immune activation.^7–9,20^ Off-the-shelf availability should therefore not be confused with simplicity. As molecular specificity increases, stability, correct assembly, pharmacokinetics, formulation, and scalable manufacturing may become limiting translational variables.^7–9^ In lupus, these constraints matter because chronic use, repeated dosing, and broad clinical deployment require molecules that are not only mechanistically plausible, but also practical to deliver.

## Therapeutic logics for SLE, including lupus nephritis

Multispecific antibodies relevant to SLE can be grouped less by molecular format than by therapeutic logic (Figure 1).^7–19^ This framing matters because dual-pathway blockers, inhibitory B-cell silencers, and T-cell engagers pursue different degrees of immune intervention.^10–14,16–19^ Treating them as a single therapeutic class risks obscuring the most important clinical question: how much immune intervention is needed, and in whom?^4,12–14,21^

**Figure 1.**
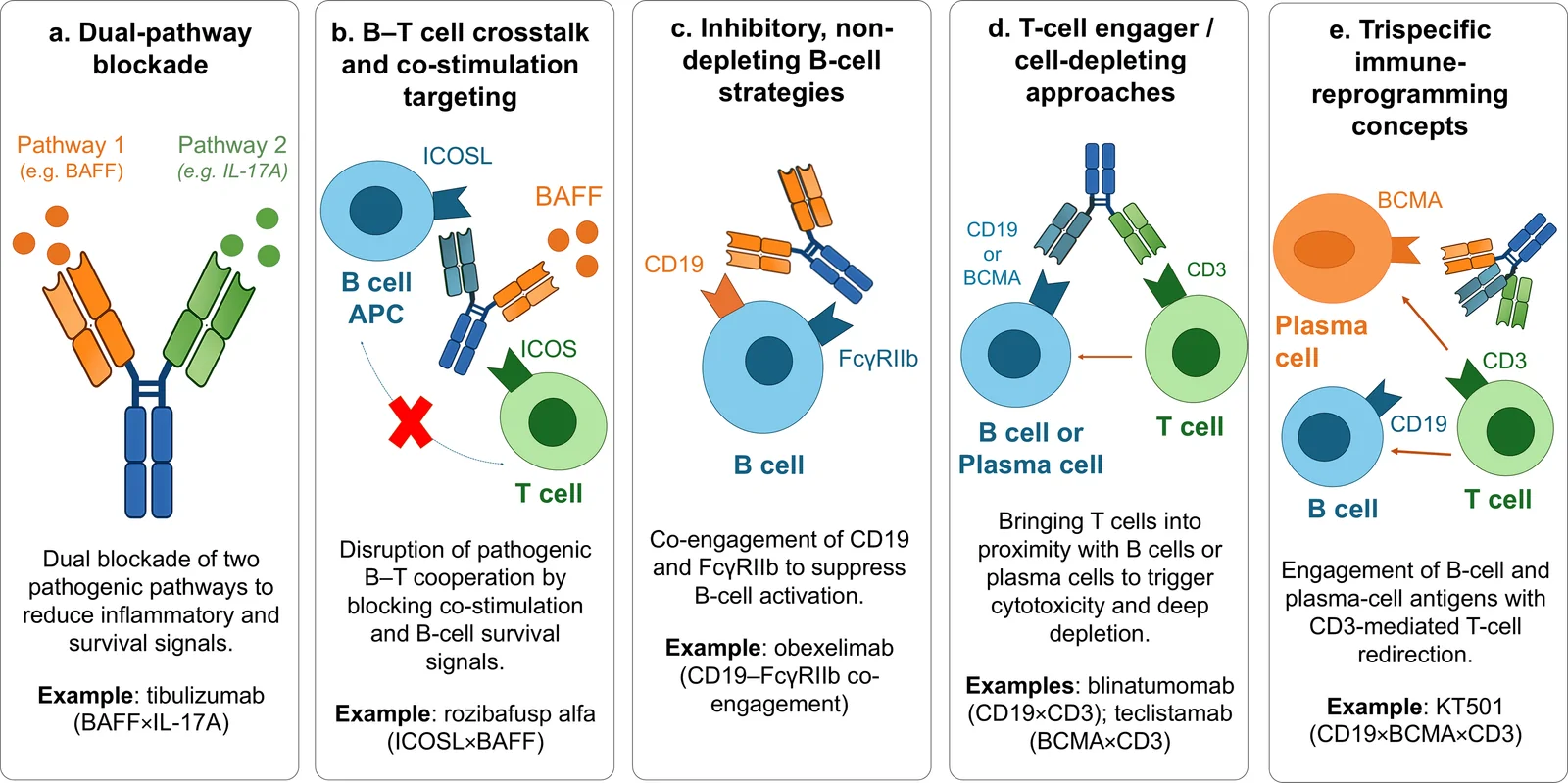
Therapeutic logics of multispecific antibody strategies relevant to SLE and lupus nephritis. Conceptual overview of the main multispecific approaches discussed in this article. (a) Dual-pathway blockade combines inhibition of two pathogenic mediators or pathways, exemplified by BAFF × IL-17A strategies.^11,15^ (b) B-T-cell crosstalk and co-stimulation targeting aims to disrupt ICOSL-ICOS-dependent T-cell help while modulating BAFF-mediated B-cell survival, exemplified by rozibafusp alfa.^10,16,21^ (c) Inhibitory, non-depleting B-cell strategies co-engage CD19 and FcγRIIb to suppress B-cell activation, exemplified by obexelimab.^12,17^ (d) T-cell engager platforms redirect endogenous T cells through CD3 toward CD19- or BCMA-expressing B-cell/plasma-cell-lineage targets, exemplified by blinatumomab and teclistamab.^13,14,19^ (e) Trispecific immune-reprogramming concepts may combine broader B-cell and plasma-cell targeting with CD3-mediated T-cell redirection, exemplified by early CD19 × BCMA × CD3 designs.^20^ The figure is intended as a mechanistic framework and does not imply clinical maturity, comparative efficacy, or therapeutic ranking. Abbreviations: BAFF, B-cell activating factor; BCMA, B-cell maturation antigen; CD, cluster of differentiation; FcγRIIb, Fc gamma receptor IIb; ICOS, inducible T-cell co-stimulator; ICOSL, inducible T-cell co-stimulator ligand; IL-17A, interleukin-17A; LN, lupus nephritis; SLE, systemic lupus erythematosus.

### Dual-pathway blockade and B-T-cell crosstalk

The most conservative use of bispecificity is dual-pathway blockade.^10,11,15,16^ The aim is not to deplete immune cells or reset autoimmunity, but to inhibit two biologically linked disease drivers within one molecule.^10,11,15,16^ This approach is attractive in SLE because B-cell survival, T-cell help, cytokine amplification, and tissue inflammation reinforce one another, and blocking one pathway may allow others to compensate.^4,10,11^

The ICOSL-BAFF axis is the clearest lupus-relevant example.^10,16^ BAFF supports B-cell survival and differentiation, whereas ICOSL participates in T-cell help and T-dependent antibody responses.^10,16^ Preclinical work with AMG 570, later known as rozibafusp alfa, showed that dual ICOSL/BAFF inhibition could outperform single-pathway inhibition in murine lupus and arthritis models.^10^ Human phase 1b data in rheumatoid arthritis subsequently confirmed pharmacodynamic engagement of both pathways, including dose-related ICOSL receptor occupancy and B-cell subset changes consistent with BAFF inhibition.^16^ However, the phase 2b lupus data are currently available only at abstract level and did not show convincing separation from standard of care.^21^ Rozibafusp therefore remains biologically plausible but clinically unproven in SLE.^10,16,21^

The ICOS-ICOSL pathway is also a model for targeting B-T-cell crosstalk.^10,16,22^ ICOS is expressed on activated T cells, whereas ICOSL is expressed on B cells and antigen-presenting cells.^10,22^ The pathway supports follicular helper T-cell biology and T-dependent antibody responses, both relevant to lupus autoimmunity.^10,22^ The randomised AMG 557 study, although testing a monospecific anti-ICOSL antibody, showed biological and clinical activity in lupus arthritis and helped validate ICOSL as a therapeutic node in human SLE.^22^ Rozibafusp extends this rationale by adding BAFF inhibition to ICOSL blockade, thereby targeting both T-cell help and B-cell survival.^10,16^

A second example is BAFF × IL-17 targeting.^11,15^ Tibulizumab, a tetravalent BAFF/IL-17A bispecific, illustrates the engineering and biological rationale of dual cytokine antagonism in autoimmunity, although it was not developed specifically as a lupus drug.^15^ More directly, a BAFF × IL-17 bispecific single-chain construct reduced autoantibodies, proteinuria, renal pathology, and inflammatory markers in MRL/lpr lupus mice.^11^ These findings support the concept that bispecific blockade can be designed around complementary pathogenic circuits in lupus, but the evidence remains preclinical.^11,15^

### Inhibitory B-cell silencing

A distinct therapeutic logic asks whether pathogenic B-cell activity can be silenced without profoundly removing the B-cell compartment.^12,17,18^ This is relevant in SLE because B cells are central to pathogenesis but also essential for immune competence.^4,12,17^ A strategy that reduces autoreactive B-cell activation while preserving part of the compartment could offer a different efficacy-safety balance from depletion-heavy approaches.^12,17^

Obexelimab is the key example.^12,17^ It is a bifunctional, non-cytolytic antibody that binds CD19 and co-engages inhibitory FcγRIIb, thereby delivering an inhibitory signal to B cells, plasmablasts, and plasma cells.^12,17^ Mechanistically, it attempts to reproduce a physiological brake on B-cell activation rather than to produce conventional cytotoxic depletion.^12,17^ Preclinical work showed that pharmacologic co-engagement of BCR-associated pathways and FcγRIIb can inhibit B-cell activation in vivo while preserving B-cell numbers.^17^

In the phase 2 SLE trial, obexelimab reduced circulating B cells by approximately 50%, but its clinical signal was nuanced.^12^ The primary endpoint was not met, whereas time to loss of improvement was prolonged and biomarker-defined subgroups appeared more likely to benefit.^12^ The lesson is not that inhibitory B-cell silencing has been proven, but that non-depleting or partially depleting approaches can generate lupus-relevant biological and clinical signals when aligned with B-cell pathway activity.^12^ Older CD22/CD20 trogocytosis-based bispecific concepts support the broader idea that lupus-oriented antibody engineering has long included attempts to disable pathogenic B-cell behaviour without simply maximising depletion.^18^

### T-cell engagers and trispecific horizons

T-cell engagers represent the most aggressive multispecific strategy because they bring CD3-expressing T cells into proximity with B-cell or plasma-cell-lineage targets, generating cytotoxic synapses and target-cell killing.^7–9,13,14,19^ In lupus, this logic is attractive because conventional therapies may leave behind autoreactive B cells, plasmablasts, or plasma-cell compartments that sustain autoantibody production.^4,13,14,23^ CD19 targeting covers a broad B-cell and plasmablast compartment, whereas BCMA targeting extends toward antibody-secreting plasma-cell-lineage populations.^13,14,23^

The direct lupus evidence is extremely limited but provocative.^13^ Teclistamab, a BCMA × CD3 bispecific approved in multiple myeloma, was reported to induce remission in a patient with highly refractory SLE and active LN.^13^ The case suggests that plasma-cell-directed T-cell engagement can reduce autoantibodies and improve nephritis, but it also shows the cost of this depth, including grade 2 cytokine release syndrome requiring tocilizumab, infections, and hypogammaglobulinaemia requiring IVIG substitution.^13^

Evidence outside lupus strengthens biological plausibility but does not establish lupus efficacy.^14,19^ In refractory rheumatoid arthritis, blinatumomab induced rapid B-cell depletion, autoantibody reduction, and clinical improvement in a small compassionate-use series.^19^ In refractory antisynthetase syndrome and systemic sclerosis, blinatumomab and teclistamab reduced target-cell populations and autoantibody levels, with clinical improvement or stabilisation, but cytokine release syndrome and infections were frequent.^14^ These observations show that the mechanism is executable in severe autoimmunity, but they do not prove safety, durability, or broad efficacy in SLE.^13,14,19^

Trispecific antibodies extend the same logic further by combining three binding specificities within one molecule.^7–9,20^ Early CD19/BCMA/CD3 designs, including KT501, suggest that broad B-cell and plasma-cell targeting with T-cell engagement is technically feasible in preclinical or abstract-level data.^20^ There is no mature lupus-specific clinical evidence, and additional specificity may add complexity, altered pharmacokinetics, immunogenicity, and excessive immune activation.^7–9,20^ Trispecifics should therefore be presented as a future horizon rather than as a near-term solution for routine SLE care.^20^

## What evidence actually exists in lupus?

The disease-specific evidence base consists mainly of one randomised phase 2 trial of an inhibitory B-cell strategy, one single-case report of a plasma-cell-directed T-cell engager in refractory SLE with active nephritis, one lupus-directed dual-pathway programme reported at abstract level, one open-label CD20/CD47 fusion-protein programme, and several preclinical or early-development concepts.^10–14,20,21,24–26^ This is enough to justify attention, but not enough to claim therapeutic maturity.^12–14,21^

Current lupus-relevant evidence and multispecific antibody programmes in clinical development are summarised in Tables 1 and 2, respectively. Obexelimab provides the most informative controlled lupus dataset to date.^12^ It tests CD19 engagement combined with FcγRIIb-mediated inhibitory signalling, a logic distinct from conventional B-cell depletion.^12,17^ In the phase 2 trial, patients with active, non-organ-threatening SLE were randomised to obexelimab or placebo after improvement during screening and withdrawal of immunosuppressants, with low-dose prednisone and hydroxychloroquine allowed.^12^ The primary endpoint, reaching week 32 without loss of improvement in the efficacy-evaluable population, was not met: 42.0% of obexelimab-treated patients versus 28.6% of placebo-treated patients achieved this endpoint (P = 0.183).^12^ A prespecified secondary analysis showed prolonged time to loss of improvement (HR 0.53, P = 0.025 in the efficacy-evaluable population), and exploratory analyses suggested greater benefit in patients with higher baseline B-cell pathway activity and in specific gene-expression clusters.^12^ These findings support further study, but they remain hypothesis-generating.^12^

**Table 1.** Current lupus-relevant evidence for multispecific antibody strategies.

Agent/strategy	Target(s)	Best available evidence	Main signal	Key caveat
Obexelimab	CD19/FcγRIIb	Randomised phase 2 SLE trial^12^	Delayed loss of improvement; biomarker signal^12^	Primary endpoint not met^12^
Rozibafusp alfa/AMG 570	ICOSL/BAFF	Preclinical SLE; RA phase 1b; SLE phase 2b abstract^10,16,21^	Dual T-B-axis blockade; target engagement^10,16^	SLE efficacy unproven^21^
IMC-002/amulirafusp alfa	CD20 mAb–SIRPα fusion protein targeting CD20/CD47^25,26^	Open-label phase Ib/II SLE data^24,26^	Deep B-cell depletion; preliminary disease-activity and biomarker signal^24^	Small, uncontrolled, abstract/registry-level data^24,26^
Teclistamab	BCMA × CD3	Single refractory SLE/LN case^13^	Deep humoral depletion; remission signal^13^	Anecdotal; CRS/infections^13^
BAFF × IL-17 constructs	BAFF/IL-17	Preclinical SLE^11^	Improved autoantibodies, proteinuria, renal inflammation^11^	No human SLE data^11^
T-cell engagers in other autoimmune diseases	CD19 × CD3; BCMA × CD3	RA, antisynthetase syndrome, systemic sclerosis^14,19^	Human proof of mechanism^14,19^	Not lupus efficacy^14,19^
Trispecific concepts	e.g. CD19/BCMA/CD3	Preclinical/abstract-level^20^	Broader immune-reprogramming concept^20^	No lupus clinical evidence^20^

Lupus-relevant evidence for selected multispecific antibody strategies. “Main signal” summarizes the most relevant biological or clinical observation available to date and should not be interpreted as established efficacy. Abbreviations: BAFF, B-cell activating factor; BCMA, B-cell maturation antigen; CRS, cytokine release syndrome; FcγRIIb, Fc gamma receptor IIb; ICOSL, inducible T-cell co-stimulator ligand; IL-17, interleukin-17; LN, lupus nephritis; RA, rheumatoid arthritis; SLE, systemic lupus erythematosus.

**Table 2.** Lupus-relevant multispecific antibody programmes in clinical development.

Programme	Target/format	Lupus-relevant population	Phase	NCT	Developmental interpretation
Regeneron LN umbrella study	Odronextamab CD20 × CD3; vonsetamig BCMA × CD3	LN	1	NCT06975787 [30]	Umbrella test of B-cell and plasma-cell-lineage T-cell engagement in LN
HB2198	Tetravalent CD19 × CD20 bispecific antibody	Moderate-to-severe active SLE, including LN and extra-renal manifestations	1	NCT07491900 [31]	Dual B-cell targeting without CD3 engagement
IMC-002/amulirafusp alfa	CD20 mAb–SIRPα fusion protein targeting CD20/CD47	Active SLE	1b/2	NCT06535412 [26]	CD20/CD47 fusion-protein strategy with preliminary lupus-directed development
YK012	CD19 × CD3 T-cell engager	Moderate-to-severe SLE	1b/2	NCT07010835 [32]	Early lupus-directed CD19 T-cell engager programme
CLN-978	Half-life-extended CD19 × CD3 T-cell engager	Moderate-to-severe SLE	1b	NCT06613360 [33]	Subcutaneous CD19-directed T-cell engager approach
GB261	CD20 × CD3 T-cell engager	Refractory seropositive SLE	1	NCT06945068 [34]	CD20-directed T-cell engagement in refractory seropositive SLE
SAR448501/DR-0201	Multispecific B-cell-depleting/myeloid-engaging platform^a^	SLE and RA	1	NCT06647069 [35]	Basket autoimmune phase 1 programme; target/format descriptor remains cautious
GR1803	BCMA × CD3 bispecific antibody^a^	SLE	1b/2a	NCT07348055 [36]	BCMA-directed T-cell engagement concept in SLE
MSC303	Subcutaneous CD20 × CD3 bispecific antibody^a^	LN and ANCA-associated GN	1b/2	NCT07000292 [37]	Subcutaneous CD20 × CD3 strategy extended to immune glomerular disease

^a^For some programmes, public registry records do not fully disclose target/format details in the same way as CD19 × CD3, CD20 × CD3, or BCMA × CD3 programmes. Descriptors for SAR448501/DR-0201, GR1803, and MSC303 should therefore be interpreted cautiously when derived from public sponsor or programme materials rather than from the SLE/LN registry entry alone.

Publicly registered lupus-relevant multispecific antibody programmes in clinical development. The table documents development activity, not efficacy. Target and format descriptors are taken from ClinicalTrials.gov records when disclosed and supplemented, where necessary, by public sponsor or programme materials; descriptors not fully disclosed in registry records should be interpreted as secondary-provenance information. Abbreviations: ANCA-GN, ANCA-associated glomerulonephritis; BCMA, B-cell maturation antigen; CD, cluster of differentiation; GN, glomerulonephritis; LN, lupus nephritis; RA, rheumatoid arthritis; SLE, systemic lupus erythematosus.

The teclistamab report represents a different type of evidence.^13^ In a patient with highly refractory SLE and active LN, BCMA × CD3 T-cell engagement was associated with deep effects on humoral autoimmunity, including reduction of anti-dsDNA antibodies, depletion of B-cell and plasma-cell-lineage compartments, improvement in nephritis and haematological manifestations, and clinical remission.^13^ This case moves the lupus discussion beyond conventional B-cell depletion toward plasma-cell-inclusive strategies, but it cannot establish reproducibility or safety at population level.^13^ The same case also captures the core safety dilemma because response occurred with grade 2 cytokine release syndrome requiring tocilizumab, pneumonia, sinusitis, and hypogammaglobulinaemia requiring IVIG substitution.^13^ These two experiences should not be interpreted as equivalent negative results. In the obexelimab trial, the treatment difference favoured obexelimab, but the study was a small proof-of-concept trial and the placebo response of 28.6% exceeded the 10% assumed in the power calculation.^12^ The primary analysis also used a reduced efficacy-evaluable population with unequal group sizes (50 obexelimab and 42 placebo), which complicated interpretation.^12^ The binary week-32 endpoint may also have been less sensitive than the time-to-event analysis.^12^ Infusion-related discontinuations, frequently associated with anti-drug antibodies, added further uncertainty, while the exposure-response relationship and biomarker-defined signals suggest that biological heterogeneity and the absence of prospective enrichment may have diluted the treatment effect.^12^ Teclistamab, by contrast, induced clinical remission in the reported patient; what remains uncertain is whether this response is reproducible and durable, and whether cytokine release syndrome, infections, and hypogammaglobulinaemia would be acceptable in a broader lupus population.^13^

Rozibafusp alfa occupies an intermediate position.^10,16,21^ Its rationale is strong because dual ICOSL/BAFF blockade is supported by preclinical lupus models and phase 1b rheumatoid arthritis data showing pharmacodynamic target engagement.^10,16^ Yet the phase 2b SLE study has been reported only at abstract level and did not show convincing separation from standard of care in a trial environment complicated by high placebo response.^21,27^ Rozibafusp therefore illustrates both the appeal and the difficulty of rational dual-pathway blockade in heterogeneous, background-treated SLE.^10,16,21,27^ Another emerging lupus-directed strategy is IMC-002/amulirafusp alfa, a CD20 mAb–SIRPα fusion protein targeting CD20/CD47.^24–26^ Preliminary open-label phase Ib/II data in moderate-to-severe active SLE suggest disease-activity and biomarker improvements with deep B-cell depletion, but the evidence remains early, uncontrolled, and available mainly at abstract or registry level.^24,26^

Preclinical BAFF × IL-17 constructs and trispecific immune-engaging platforms are still earlier.^11,20^ The BAFF × IL-17 model supports plausibility of dual cytokine blockade in lupus-like disease, whereas CD19/BCMA/CD3 trispecific designs suggest that broad B-cell and plasma-cell targeting may become technically feasible.^11,20^ These signals should define direction, not clinical practice.^11,20^

The lupus nephritis evidence is especially thin.^12,13,21^ Obexelimab was tested in non-organ-threatening SLE rather than active LN, teclistamab included active nephritis in a single patient, and rozibafusp has not provided a peer-reviewed renal efficacy dataset sufficient for clinical inference.^12,13,21^ This is a major gap because LN is one of the settings where deeper B-cell or plasma-cell-directed strategies may be most attractive and where safety, background therapy, and endpoint selection are most consequential.^2,4,13,23,27^ The current lupus evidence therefore supports neither dismissal nor premature adoption.^12–14,21^ The signal is real, but the role remains undefined.^12–14,21^

## Lessons from other autoimmune diseases

Evidence from other autoimmune diseases should be used carefully because rheumatoid arthritis, systemic sclerosis, antisynthetase syndrome, and inflammatory myopathies do not predict efficacy across SLE manifestations, including LN.^14,16,19^ They differ in target organs, tissue immunology, autoantibody biology, background treatment, and outcome measures.^14,16,19^ Their value is narrower but important: they show that some multispecific mechanisms are biologically executable in humans with autoimmune disease.^14,16,19^

Rheumatoid arthritis has provided two useful signals.^16,19^ The phase 1b study of rozibafusp alfa showed that dual ICOSL/BAFF blockade can achieve pharmacodynamic engagement in humans, including high ICOSL receptor occupancy and B-cell subset changes consistent with BAFF inhibition.^16^ Compassionate-use blinatumomab in multidrug-resistant rheumatoid arthritis showed that CD19×CD3 T-cell engagement can deplete B cells, reduce autoantibodies, and induce rapid clinical improvement outside oncology, although the series was very small and toxicity conclusions remain preliminary.^19^

Systemic sclerosis and antisynthetase syndrome provide a more severe systemic-autoimmunity bridge.^14^ In a compassionate-use series, blinatumomab was used in refractory antisynthetase syndrome and teclistamab in refractory systemic sclerosis.^14^ Treatment reduced target-cell populations in affected tissues, lowered autoantibody titres, and was associated with clinical improvement or stabilisation of myositis, skin fibrosis, and interstitial lung disease.^14^ The same experience also reported cytokine release syndrome, including grade 3 events, and respiratory infections requiring antibiotics.^14^ This pattern is highly relevant for SLE, where many patients already carry infection risk from disease activity, glucocorticoids, nephritis, renal impairment, hypogammaglobulinaemia, or prior immunosuppression.^4,13,14^

A further transferable lesson is that induction and maintenance may need to be separated.^14^ In the connective tissue disease experience, rituximab maintenance after T-cell engager induction appeared to help prolong disease control, even in patients previously unresponsive to rituximab alone.^14^ This observation should not be overgeneralised, but it raises an important lupus question about how to maintain disease control after deep depletion without prolonging immune suppression unnecessarily.^14^ Non-lupus autoimmune data should therefore inform lupus development, not substitute for lupus-specific trials.^12–14,21,27^

## Safety, patient selection, and trial-design barriers

The development of multispecific antibodies in lupus will depend as much on safety and patient selection as on molecular efficacy.^12–14,21^ The class contains agents with very different depths of immune intervention, so a dual-pathway blocker, an inhibitory B-cell silencer, and a CD3-based T-cell engager should not be expected to share the same risk profile.^10–14^ In practice, the key trade-off is how deeply the immune system is pushed, and how much infection risk, hypogammaglobulinaemia, or delayed immune recovery is acceptable for that depth of intervention.^10–14,16–19^

The first safety issue is on-target immune suppression.^12–14^ If a therapy suppresses B-cell survival, inhibits B-cell activation, or removes plasma cells, it may also impair protective humoral immunity.^12–14^ The clinical implications include infections, hypogammaglobulinaemia, impaired vaccine responses, and uncertain long-term immune reconstitution.^13,14^ These concerns may be more modest for some inhibitory or dual-blocking approaches, but they become central for T-cell engagers and plasma-cell-directed strategies.^12–14,16^ The teclistamab lupus case illustrates this clearly because clinical response was accompanied by hypogammaglobulinaemia, IVIG substitution, and infectious complications.^13^

Cytokine release syndrome is the second major barrier.^13,14,19^ It is most relevant for CD3-based engagers, where therapeutic efficacy depends on T-cell activation.^13,14,19^ In lupus, tolerance for cytokine release syndrome is lower than in oncology because patients may already have organ involvement, infection risk, or steroid exposure.^4,13,14^ Autoimmune data remain limited, but cytokine release syndrome has already appeared in the teclistamab SLE case and the connective tissue disease series.^13,14^ Step-up dosing, early cytokine monitoring, infection surveillance, and clear rescue protocols will therefore be central to feasibility.^13,14^

Whether autoimmune indications will require lower doses than those used in malignancy is not yet established. Early experience with T-cell engagers suggests that reducing cumulative exposure or treatment duration may be at least as important as lowering the individual dose. In refractory rheumatoid arthritis, blinatumomab was deliberately administered at low dose and reduced duration, using 9 µg/day for two 5-day courses.^19^ By contrast, in the reported SLE case, teclistamab reached the full 1.5 mg/kg dose after step-up dosing, but treatment was limited to five injections.^13^ These observations support disease-specific dose-finding and careful minimisation of exposure rather than assuming a uniform dose reduction across autoimmune indications.^13,19^

Reversibility is a practical advantage of off-the-shelf multispecific antibodies over cellular therapy.^7–9,13,14,28,29^ Antibody-based therapies can usually be interrupted, dose-adjusted, repeated, or discontinued.^7–9,13,14^ This matters in lupus, where disease activity fluctuates and treatment intensity often changes over time.^4,27^ However, reversibility at the drug level does not guarantee rapid immune-system recovery, because a short course of a powerful T-cell engager may produce prolonged B-cell or plasma-cell depletion and delayed immunoglobulin recovery.^13,14^ A further uncertainty is whether endogenous T-cell fitness, which may be altered by severe disease and prior immunosuppression, will limit the efficacy or tolerability of CD3-based engagers in heavily pretreated lupus patients.^13,14,19^

Patient selection is likely to determine success or failure.^12,21,27^ Unselected SLE has repeatedly diluted therapeutic signals in clinical trials, and multispecific antibodies are unlikely to escape this problem.^12,21,27^ Obexelimab suggests that biological enrichment may matter because patients with stronger baseline B-cell pathway signatures appeared more likely to benefit, although this remains exploratory.^12^ Dual ICOSL/BAFF blockade may fit patients with active T-B-cell collaboration and persistent humoral activity, whereas T-cell engagers may be more plausible in severe refractory antibody-driven disease where the immune cost is justified.^10,12–14,16,21^

Trial-design challenges differ between LN and non-renal SLE.^1–3,12,21,27^ LN trials benefit from organ-specific and comparatively objective measures, particularly urine protein-to-creatinine ratio and estimated glomerular filtration rate, which are incorporated into composite renal-response endpoints.^2^ Kidney biopsy is central to disease classification and can provide additional histological information in selected studies, but is not usually the primary efficacy endpoint. In non-renal SLE, the Systemic Lupus Erythematosus Responder Index 4 (SRI-4) and the British Isles Lupus Assessment Group-based Composite Lupus Assessment (BICLA) are established composite outcomes and have supported successful phase 3 programmes.^1,3^ The remaining difficulty is therefore not the absence of validated endpoints, but heterogeneity across organ domains, background therapy and glucocorticoid tapering, placebo response, and the possibility that distinct multispecific mechanisms produce different response kinetics.^12,21,27^ A dual-pathway blocker may gradually reduce inflammatory and serological activity, an inhibitory B-cell strategy may delay flare or loss of improvement, and a T-cell engager may produce rapid depletion and a more abrupt clinical signal.^10–14^

Background therapy remains an important source of confounding in both renal and non-renal trials.^12,21,27^ Corticosteroids and immunosuppressants can obscure incremental benefit, while steroid tapering can create apparent loss of response that reflects withdrawal rather than drug failure.^12,27^ The obexelimab trial attempted to manage this issue through clinical improvement before randomisation and immunosuppressant withdrawal, illustrating how sensitive lupus outcomes are to background-treatment rules.^12^ The rozibafusp phase 2b experience also highlights the challenge because high placebo response and optimised standard of care may obscure separation even for a plausible mechanism.^21,27^ Adaptive and enrichment designs, such as those described for rozibafusp and efavaleukin alfa, may therefore be essential tools for future lupus studies.^27^

Taken together, these safety, selection, and design constraints mean that clinical placement will depend less on molecular class alone than on phenotype, therapeutic goal, and acceptable immune risk.

## Where could multispecific antibodies fit?

Multispecific antibodies should not currently be imagined as first-line therapies for unselected SLE.^12–14,21^ Their most plausible role is in a stratified treatment landscape where immune-intervention intensity is matched to disease biology, severity, prior treatment failure, and acceptable risk.^4,12–14,21^ Belimumab and anifrolumab are established targeted biologics in lupus, and obinutuzumab has added an approved anti-CD20 option for active LN in relevant jurisdictions; multispecific antibodies must therefore justify themselves by solving problems that current monospecific agents do not reliably solve.^1–6^

The rationale for using a multispecific rather than another approved monospecific agent would therefore need to be explicit: simultaneous modulation of two biologically linked pathways, broader targeting of B-cell and plasma-cell compartments inadequately controlled by current therapy, or off-the-shelf, dose-adjustable immune engagement as an alternative to cellular therapy. Figure 2 summarises a proposed clinical-development framework for these different settings.^7–14,16–19,28,29^

**Figure 2.**
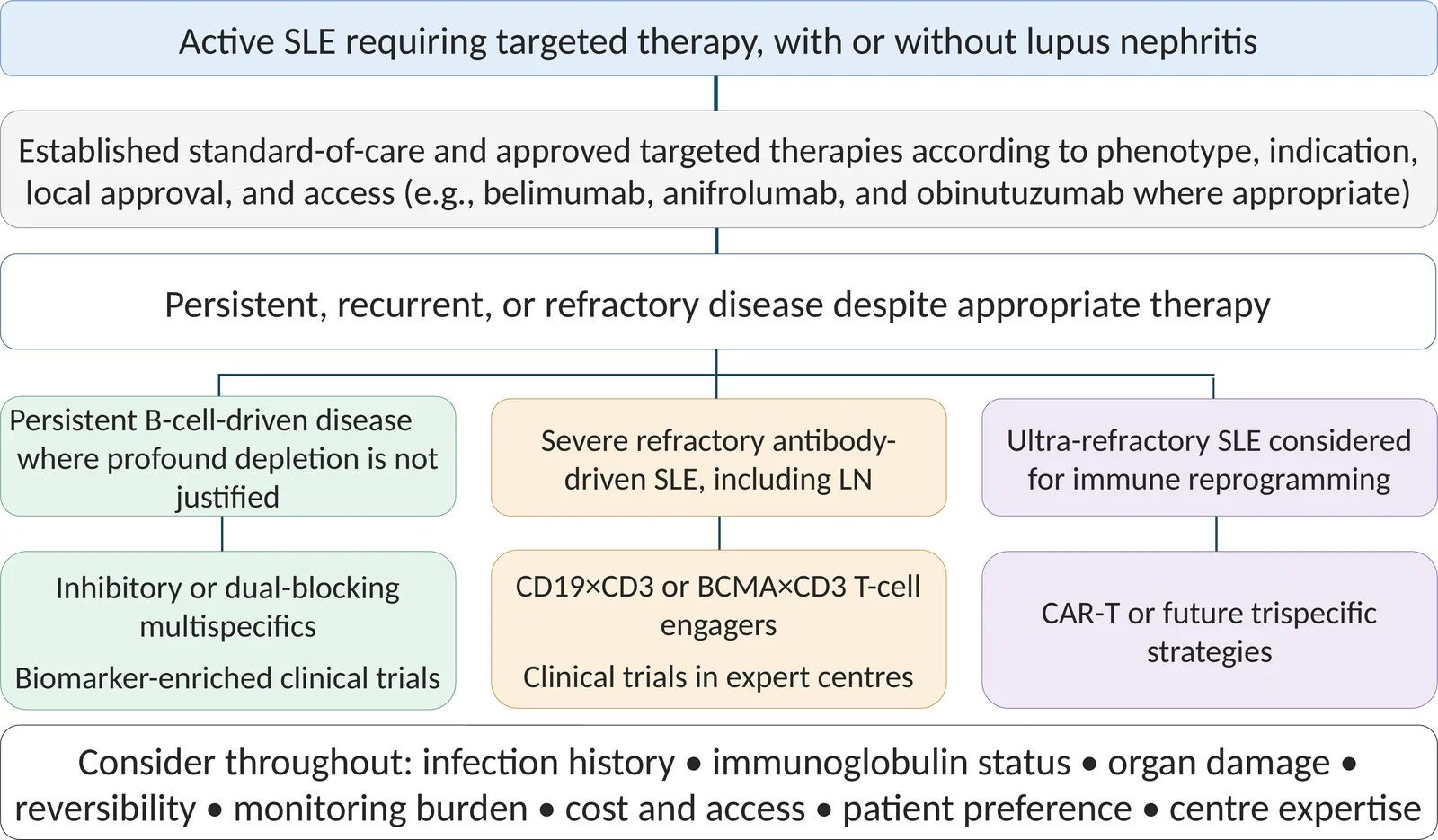
Conceptual clinical positioning of multispecific antibody strategies in SLE and lupus nephritis. This figure presents a conceptual clinical-development framework and does not constitute a treatment recommendation. Established standard-of-care and approved targeted therapies should remain the initial options according to disease phenotype, indication, local approval, and access. In patients with persistent, recurrent, or refractory disease despite appropriate therapy, inhibitory or dual-blocking multispecifics may be investigated, preferably in biomarker-enriched clinical trials, for persistent B-cell-driven disease when profound depletion is not justified. CD19 × CD3 or BCMA × CD3 T-cell engagers may be investigated in expert centres for severe refractory antibody-driven SLE, including LN. CAR-T or future trispecific strategies may be considered in highly selected patients with ultra-refractory SLE in whom immune reprogramming is being pursued. These categories are illustrative and not necessarily sequential. Infection history, immunoglobulin status, organ damage, reversibility, monitoring burden, cost and access, patient preference, and centre expertise should be considered throughout. Abbreviations: BCMA, B-cell maturation antigen; CAR-T, chimeric antigen receptor T-cell therapy; CD, cluster of differentiation; LN, lupus nephritis; SLE, systemic lupus erythematosus.

Inhibitory and dual-blocking multispecifics may be investigated after an inadequate response to established biologics, before considering deeply depleting T-cell engagers or cellular therapies.^10,11,12,16,17,21^ Obexelimab-like approaches may be relevant for persistent B-cell-driven activity when functional restraint is preferable to deep depletion.^12,17^ Dual ICOSL/BAFF blockade, if future data support efficacy, could fit patients in whom T-B-cell collaboration and B-cell survival remain active despite standard therapy, but before plasma-cell-directed depletion becomes the only rational option.^10,16,21^ These strategies may offer integrated immunomodulation without immediately entering the toxicity territory of T-cell engagers.^10–14,16^

T-cell engagers belong in a different part of the algorithm.^13,14,19^ Their plausible use is not broad outpatient lupus control, but highly refractory antibody-driven disease, including selected patients with refractory LN or other severe relapsing manifestations of SLE after failure of conventional immunosuppression and approved biologics.^13,14^ In these settings, the therapeutic goal may shift from incremental disease suppression to deeper interruption of pathogenic humoral immunity.^13,14^ The teclistamab case shows why this is conceptually attractive, but also why it should remain restricted to expert settings until better evidence is available.^13^

A separate issue is whether deep depletion, if effective, should be followed by a less intensive maintenance strategy. Experience in refractory connective tissue diseases suggests that induction and maintenance may not be equivalent phases of treatment, but analogous lupus strategies would require explicit monitoring of immunoglobulin recovery, infections, and retreatment thresholds.^14^

The comparison with CAR-T cell therapy is unavoidable.^23,28,29^ CAR-T currently provides the strongest early signal that deep immune reprogramming may be possible in severe refractory SLE, including CD19-directed and dual CD19/BCMA approaches.^23,28,29^ This efficacy signal sets a high bar for antibody-based multispecifics, but it is not toxicity-free: CAR-T protocols require lymphodepletion, and the dual CD19/BCMA phase 1 trial reported grade 3 or higher neutropenia in all treated patients.^23^ Antibody-based multispecifics have different practical attributes: they are off-the-shelf, do not require leukapheresis or individualised manufacturing, avoid ex vivo cell expansion, and can usually be dose-adjusted or discontinued.^7–9,13,14^ These features may allow them to occupy a space between conventional biologics and CAR-T if efficacy proves sufficient.^13,14,28,29^

Cost and access will also influence clinical placement. Most lupus-directed multispecific programmes remain investigational, so acquisition prices and lupus-specific cost-effectiveness data are not yet available. Although off-the-shelf antibodies avoid leukapheresis and individualised manufacturing, they should not be assumed to be affordable: CD3-based engagers may require step-up dosing, close monitoring for cytokine release syndrome and infection, immunoglobulin surveillance or replacement, and delivery in experienced centres.^13,14^ These requirements may be particularly restrictive in resource-limited health systems. Future development should therefore evaluate total treatment burden and cost-effectiveness alongside efficacy and safety.

The future is therefore more likely to be a continuum than a single dominant advanced platform.^4,12–14,21^ Patients with persistent B-cell-driven disease may be candidates for inhibitory or dual-blocking multispecifics, whereas those with severe refractory antibody-driven disease may be considered for T-cell engagers or plasma-cell-directed strategies in expert centres.^10–14,16,17,19^ Patients requiring the deepest attempt at immune reset may be evaluated for CAR-T or related cellular approaches.^23,28,29^ These boundaries will depend on trial data, biomarkers, safety experience, and access.^4,12–14,21,27^

## Conclusion

Multispecific antibodies represent more than a technical extension of monoclonal antibody therapy in SLE.^7–9^ Their importance lies in the possibility of aligning therapeutic architecture with lupus biology: blocking linked pathways, interrupting B-T-cell collaboration, delivering inhibitory signals to B cells, or redirecting effector T cells against B-cell and plasma-cell compartments.^10–14,16–19^ This conceptual shift is timely because current biologics have validated targeted therapy in lupus, but have not eliminated relapse, incomplete response, steroid dependence, or refractory organ disease.^1–4^

These are still early days in the development of bispecific and trispecific antibodies for lupus.^12–14,21^ Obexelimab provides the most informative randomised lupus dataset, but its phase 2 trial did not meet the primary endpoint, and its biomarker signals remain hypothesis-generating.^12^ Teclistamab offers a provocative proof of principle in refractory SLE with active nephritis, but only as a single case and with substantial immune toxicity.^13^ Rozibafusp alfa illustrates the appeal of rational dual-pathway blockade, but also the difficulty of demonstrating added clinical value in heterogeneous, background-treated SLE.^10,16,21^ Trispecific approaches are even less mature and should be viewed as a future horizon rather than as a current therapeutic option.^20^

The next step is to ask which multispecific mechanism fits which lupus phenotype.^4,12–14,21^ Some patients may need broader pathway modulation, others may require B-cell silencing, and a smaller subset with severe refractory antibody-driven disease may justify deeper B-cell or plasma-cell-directed depletion.^10–14,16,17,18,19^ If future studies can match mechanism to patient biology, manage immune cost, and use trial designs suited to lupus heterogeneity, multispecific antibodies may become an important bridge between conventional biologics and cellular immune reprogramming.^12–14,21,23,27–29^

## Data Availability

No new data were generated or analysed for this article. All sources are cited in the manuscript or are publicly available registry records.*

## References

[1] Navarra SV Guzman RM Gallacher AE , et al. Efficacy and safety of belimumab in patients with active systemic lupus erythematosus: a randomised, placebo-controlled, phase 3 trial. Lancet 2011; 377: 721–731. 10.1016/S0140-6736(10)61354-2 21296403

[2] Furie R Rovin BH Houssiau F , et al. Two-year, randomized, controlled trial of belimumab in lupus nephritis. N Engl J Med 2020; 383: 1117–1128. 10.1056/NEJMoa2001180 32937045

[3] Morand EF Furie R Tanaka Y , et al. Trial of anifrolumab in active systemic lupus erythematosus. N Engl J Med 2020; 382: 211–221. 10.1056/nejmoa1912196 31851795

[4] Guo C Lei Z Zhao L , et al. Precision immunotherapies for systemic lupus erythematosus: from pathogenic targets to translational horizons. Autoimmun Rev. 2026; 25: 104029. 10.1016/j.autrev.2026.104029 41812738

[5] Genentech, Inc . Gazyva (obinutuzumab) prescribing information. Genentech, Inc, 2025.

[6] European Medicines Agency . Gazyvaro: EPAR product information. European Medicines Agency, 2026.

[7] Borrelli R Brussino L Negrini S , et al. From molecular engineering to clinical applications: the expanding role of bispecific antibodies in onco-immunology. Pharmacol Res 2025; 221: 108000. 10.1016/j.phrs.2025.108000 41110778

[8] Fine J Meksiriporn B Tan J , et al. Mechanism-driven design of multispecific antibodies for targeted disease treatment. Annu Rev Chem Biomol Eng 2024; 15: 105–138. 10.1146/annurev-chembioeng-100522-102155 38277673

[9] Zhao Q . Bispecific antibodies for autoimmune and inflammatory diseases: clinical progress to date. BioDrugs 2020; 34: 111–119. 10.1007/s40259-019-00400-2 31916225

[10] Zhang M Lee F Knize A , et al. Development of an ICOSL and BAFF bispecific inhibitor AMG 570 for systemic lupus erythematosus treatment. Clin Exp Rheumatol 2019; 37: 906–914. 30789152

[11] Xin C Zhou J Chen Y , et al. Design and development of a bispecific antibody targeting BAFF and IL-17 for systemic lupus erythematosus treatment. BMC Biol 2025; 23: 283. 10.1186/s12915-025-02398-2 PMC12482122 41024082

[12] Merrill JT Guthridge J Smith M , et al. Obexelimab in systemic lupus erythematosus with exploration of response based on gene pathway co-expression patterns: a double-blind, randomized, placebo-controlled, phase 2 trial. Arthritis Rheumatol 2023; 75: 2185–2194. 10.1002/art.42652 37459248

[13] Alexander T Kronke J Cheng Q , et al. Teclistamab-induced remission in refractory systemic lupus erythematosus. N Engl J Med 2024; 391: 864–866. 10.1056/NEJMc2407150 39231352

[14] Dusing C Gyorfi AH Stutz AN , et al. Bispecific T cell engagers for treatment-refractory autoimmune connective tissue diseases. Nat Med 2026; 32: 1530–1542. 10.1038/s41591-026-04238-4 PMC13099420 41714747

[15] Benschop RJ Chow CK Tian Y , et al. Development of tibulizumab, a tetravalent bispecific antibody targeting BAFF and IL-17A for the treatment of autoimmune disease. mAbs 2019; 11: 1175–1190. 10.1080/19420862.2019.1624463 PMC6748573 31181988

[16] Abuqayyas L Cheng LE Teixeira Dos Santos M , et al. Safety and biological activity of rozibafusp alfa, a bispecific inhibitor of inducible costimulator ligand and B cell activating factor, in patients with rheumatoid arthritis: results of a phase 1b, randomized, double-blind, placebo-controlled, multiple ascending dose study. ACR Open Rheumatol 2022; 4: 903–911. 10.1002/acr2.11487 PMC9555197 35899378

[17] Chu SY Pong E Bonzon C , et al. Inhibition of B cell activation following in vivo co-engagement of B cell antigen receptor and Fcγ receptor IIb in non-autoimmune-prone and SLE-prone mice. J Transl Autoimmun 2021; 4: 100075. 10.1016/j.jtauto.2020.100075 PMC7773957 33409482

[18] Rossi EA Chang CH Goldenberg DM . Anti-CD22/CD20 bispecific antibody with enhanced trogocytosis for treatment of lupus. PLoS One 2014; 9: e98315. 10.1371/journal.pone.0098315 PMC4026529 24841238

[19] Bucci L Hagen M Rothe T , et al. Bispecific T cell engager therapy for refractory rheumatoid arthritis. Nat Med 2024; 30: 1593–1601. 10.1038/s41591-024-02964-1 38671240

[20] Bao M Wang J Zhao J , et al. KT501, a CD19/BCMA/CD3 trispecific antibody, leads to rapid and deep B-cell depletion with well-tolerated safety. Arthritis Rheumatol. 2025; 77(Suppl 9): Abstract 0012.

[21] Barbar-Smiley F Tang F Garces S , et al. Rozibafusp alfa in patients with active systemic lupus erythematosus: results of a Bayesian adaptive phase 2b, randomized, double-blind, placebo-controlled, dose-ranging study. J Rheumatol 2025; 52(Suppl 1): 73–74. 10.3899/jrheum.2025-0390.PT014

[22] Cheng LE Amoura Z Cheah B , et al. Brief report: a randomized, double-blind, parallel-group, placebo-controlled, multiple-dose study to evaluate AMG 557 in patients with systemic lupus erythematosus and active lupus arthritis. Arthritis Rheumatol 2018; 70: 1071–1076. 10.1002/art.40479 PMC6032945 29513931

[23] Feng J Huo D Hong R , et al. Co-infusion of CD19-targeting and BCMA-targeting CAR-T cells for treatment-refractory systemic lupus erythematosus: a phase 1 trial. Nat Med. 2025; 31: 3725–3736. 10.1038/s41591-025-03937-8 40993243

[24] Yao H Tian W Zheng Q , et al. IMC-002 (IMM0306), a first-in-class bi-specific fusion protein, demonstrates improvements in systemic lupus erythematosus disease activity measures and biomarkers in patients with moderate to severe active SLE in the open-label phase 1b/2 study. Arthritis Rheumatol. 2025; 77(Suppl 9): Abstract 2464.

[25] Yang J Song Y Zhou K , et al. Safety and efficacy of amulirafusp alfa (IMM0306), a fusion protein of CD20 monoclonal antibody with the CD47 binding domain of SIRPα, in patients with relapsed or refractory B-cell non-Hodgkin lymphoma: a phase 1/2 study. J Hematol Oncol. 2024; 17: 123. 10.1186/s13045-024-01646-2 PMC11657391 39696680

[26] ClinicalTrials.gov . A study of IMC-002 for the treatment of active systemic lupus erythematosus. ClinicalTrials.gov identifier: NCT06535412. U.S. National Library of Medicine. Available at: https://clinicaltrials.gov/study/NCT06535412

[27] Garces S Karis E Merrill JT , et al. Improving resource utilisation in SLE drug development through innovative trial design. Lupus Sci Med 2023; 10: e000890. 10.1136/lupus-2022-000890 PMC10373732 37491104

[28] Mackensen A Muller F Mougiakakos D , et al. Anti-CD19 CAR T cell therapy for refractory systemic lupus erythematosus. Nat Med 2022; 28: 2124–2132. 10.1038/s41591-022-02017-5 36109639

[29] Muller F Taubmann J Bucci L , et al. CD19 CAR T-cell therapy in autoimmune disease: a case series with follow-up. N Engl J Med 2024; 390: 687–700. 10.1056/NEJMoa2308917 38381673

[30] ClinicalTrials.gov . Dose escalation study with bispecific antibodies in adult patients with lupus nephritis. clinicalTrials.gov identifier: NCT06975787. U.S. national library of medicine. Available at: https://clinicaltrials.gov/study/NCT06975787

[31] ClinicalTrials.gov . A phase 1 study of HB2198 in participants with moderately to severely active systemic lupus erythematosus. ClinicalTrials.gov identifier: NCT07491900. U.S. National Library of Medicine. Available at: https://clinicaltrials.gov/study/NCT07491900

[32] ClinicalTrials.gov . Study of YK012 in moderate to severe systemic lupus erythematosus. ClinicalTrials.gov identifier: NCT07010835. U.S. National Library of Medicine. Available at: https://clinicaltrials.gov/study/NCT07010835

[33] ClinicalTrials.gov . A study of CLN-978, a subcutaneously administered CD19-directed T cell engager, in subjects with systemic lupus erythematosus. ClinicalTrials.gov identifier: NCT06613360. U.S. National Library of Medicine. Available at: https://clinicaltrials.gov/study/NCT06613360

[34] ClinicalTrials.gov . An open-label study of GB261 in refractory seropositive systemic lupus erythematosus. ClinicalTrials.gov identifier: NCT06945068. U.S. National Library of Medicine. Available at: https://clinicaltrials.gov/study/NCT06945068

[35] ClinicalTrials.gov . A study to evaluate the safety and activity of SAR448501/DR-0201 in patients with autoimmune rheumatic diseases. ClinicalTrials.gov identifier: NCT06647069. U.S. National Library of Medicine. Available at: https://clinicaltrials.gov/study/NCT06647069

[36] ClinicalTrials.gov . A study of GR1803 in systemic lupus erythematosus. ClinicalTrials.gov identifier: NCT07348055. U.S. National Library of Medicine. Available at: https://clinicaltrials.gov/study/NCT07348055

[37] ClinicalTrials.gov . MSC303 subcutaneous injection for the treatment of immunologic glomerular diseases. ClinicalTrials.gov identifier: NCT07000292. U.S. National Library of Medicine. Available at: https://clinicaltrials.gov/study/NCT07000292

